# Dietary administration with hydrolyzed silk sericin improves the intestinal health of diabetic rats

**DOI:** 10.3389/fmicb.2023.1074892

**Published:** 2023-03-07

**Authors:** Wenlin Zhou, Yujie Weng, Qian Liu, Chonglong Wang, Yu-Qing Zhang, Xing Zhang, Aihong Ye

**Affiliations:** ^1^Institute of Sericulture and Tea, Zhejiang Academy of Agricultural Sciences, Hangzhou, China; ^2^Department of Biological Sciences, School of Biology and Basic Medical Sciences, Soochow University, Suzhou, China

**Keywords:** sericin, type II diabetes, gut microbiota, health, rats

## Abstract

Type II diabetes (T2D) is a global epidemic disease with an increased incidence and prevalence. Gut microbiota plays an important role in controlling T2D development. Dietary administration of prebiotics, probiotics, and drugs, including metformin, showed the regulatory impact on the change of gut microbiota, which is associated with the improvement of glucose tolerance. In this study, silk sericin was manufactured into hydrolyzed sericin peptide (HSP) powders as a dietary additive to investigate the effect on the gut microbiota of T2D model rats. The results indicated that the HSP-augmented dietary administration lowers the fast glucose level of diabetic rats, and HSP augmentation induces a change in the gut microbiota composition of T2D model rats toward the normal rats. Some key taxa, including *Lactobacillus gasseri*, were suggested to be involved in controlling T2D development. This finding provides new insight into developing sericin as functional food or therapeutic prebiotics against T2D in clinical practice.

## 1. Introduction

Type II diabetes (T2D), accounting for approximately 90% of all diabetes, is a globally prevalent disease associated with obesity and an unhealthy lifestyle. It is characterized by insulin deficiency, hyperglycemia, and metabolic disorder of many organs, resulting in a high risk of T2D mortality and morbidity (Zimmet et al., 2001). The estimated prevalence of diabetes in adults (aged 20–79 years) has tripled from 151 million in 2000 to 463 million in 2019, according to International Diabetes Federation reports (9th edition). The population with diabetes will rise by 10.2% to 578 million by 2030 if no sufficient actions are implemented. However, T2D can be effectively managed by the support and adoption of healthy lifestyles in combination with as-required medication, such as insulin and glucose absorption inhibitors. In the past decade, it has been demonstrated that gut microbial dysbiosis contributes to the risk of developing obesity and diabetes (Tilg and Moschen, 2014; Wang and Jia, 2016). The gut microbiota is a complex ecosystem of microorganisms in the intestinal tract, which can affect host physiology and serves as the therapeutic route for antidiabetic medications (Wu et al., 2017). A cohort analysis of the gut microbiota of patients with T2D indicated significantly reduced proportions of the phylum Firmicutes and the class Clostridia compared with those of healthy controls (Larsen et al., 2010). Opportunistic pathogens such as *Bacteroides, Clostridiales, Escherichia coli*, and sulfate-reducing species *Desulfovibrio* were often enriched in patients with diabetes (Qin et al., 2012). Gut microbiota can be an active site of the antidiabetic drug metformin, which alters microbiota composition and improves glucose tolerance (Wu et al., 2017; Foretz et al., 2019). Metformin is reported to increase short-chain fatty acid (SCFA)-producing bacteria and *Lactobacillus* species and decrease *Bacteroides fragilis* (Forslund et al., 2015; de la Cuesta-Zuluaga et al., 2017; Bauer et al., 2018). The metabolic functions of microbiota and their interaction with the host metabolism can be reconstructed upon the composition alternation, e.g., bile acid homeostasis, glucagon-like peptide 1 (GLP1) secretion, and activation of the gut–brain–liver neuronal axis. Dietary administration of prebiotics, probiotics, and drugs appears to have a beneficial impact on insulin resistance in clinical or animal T2D models (Belizário and Napolitano, 2015; Salgaço et al., 2019; Rodrigues et al., 2021), although further evaluation is required in more individuals with diabetes (Bordalo Tonucci et al., 2017). Oral intake of probiotics or berberine has been reported to alter microbial bile acid metabolism and improve glycemic control, which is ascribed to the resultant changes in gut microbiota (Zhang et al., 2020). In any case, the manipulation of gut microbiota by dietary administration can be a readily adopted approach to control and ameliorate diabetes (Ghorbani et al., 2021).

Sticky sericin coats the core fibroin and is removed as waste in silk processing (Aramwit et al., 2012). It is a mixture of macromolecule polypeptides with a molecular mass of 10–300 kDa and constitutes more than 25% of the total cocoon weight (Cao and Zhang, 2016). Sericin shows various biological activities, including antioxidation, inhibition of tyrosinase, and protection against alcohol-induced liver and gastric injuries (Li et al., 2008; Cherdchom et al., 2021; Suzuki et al., 2022). Moreover, dietary sericin lowers the levels of triglyceride and cholesterol in rats fed with a high-fat diet (Seo et al., 2011). In this study, sericin diets were given to diabetic rats to assess the improvement of intestinal health through the alternation of gut microbiota. The result will be useful in the development of sericin as a functional food or a therapeutic agent against T2D.

## 2. Materials and methods

### 2.1. Preparation of hydrolyzed sericin peptide powders

**Figure 1:**
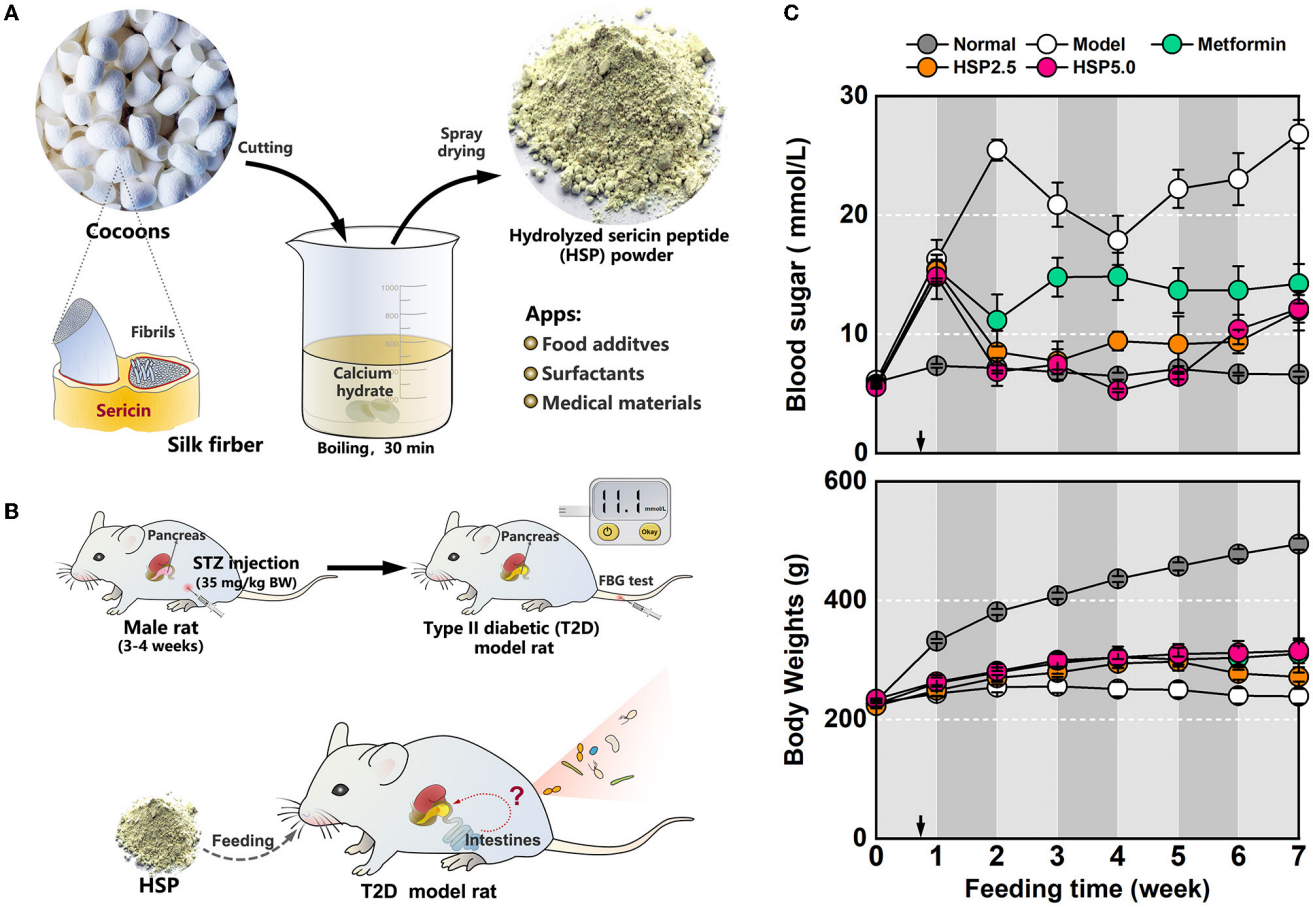
Dietary administration with hydrolyzed sericin peptide (HSP) in T2D model rats. **(A)** Schematic of HSP processing. **(B)** T2D model preparation and HSP dietary administration. **(C)** Measurement of body weights and FBG levels. The measured values are presented as mean ± standard error.

Hydrolyzed sericin peptide (HSP) powders were prepared according to a modified method described previously (Zhang M. et al., 2019; Dong et al., 2020). In brief, the cocoon shells were weighted and soaked in 0.025% (w/v) calcium hydroxide at a 1:90 bath ratio (w/v). The degumming was processed using a 30-min boiling treatment, which was conducted twice to improve sericin recovery. Then, the degumming solutions were condensed through rotary evaporation under the protection of negative pressure. The condensed solution was neutralized with sulfuric acid, and the resultant calcium sulfate precipitate was removed by centrifugation. The collected supernatant was finally dried by using a vacuum-freezing spray dryer (Figure 1A).

### 2.2. Animals

Male Sprague–Dawley rats weighing approximately 200 g were maintained in dim cyclic light (20–40 lux, 12-h light:12-h dark/light cycle) at 20–25 °C and 50–80% humidity, and the water and diets were provided *ad libitum* unless otherwise noted. The T2D model rats were intraperitoneally injected with streptozotocin (STZ) (Dong et al., 2020; Wei and Weng, 2022). The rats fasted overnight before the injection, and the injection dose was administered for 3 days, at a low dose of 35 mg/kg body weight per day. Normal diets were provided during the injection period, and a high-fat and high-sugar diet was consequently fed to the administrated rats for 5 days after injection. Five days after the first injection, tail vein blood was collected to measure the fasting blood sugar (FBG) level; rats with an FBG level higher than 11.1 mmol/L were considered T2D model rats (Figure 1B). The T2D model rats were randomly divided into four dietary groups (*n* = 4–7) for the experiment. All animal procedures were approved by the International Animal Welfare Committee and the Animal Experimental Operations and Ethics Committee of Soochow University (Animal License No. 201802A128).

### 2.3. Diets

The rats of the normal group (Normal) and one T2D model rat group (Model) were fed with irradiated standard diets only as the positive and negative controls, respectively. The rats from two T2D model groups (HSP2.5 and HSP5.0) were fed with standard diets augmented with either 2.5% (w/w) or 5.0% (w/w) HSP powders, respectively. The T2D model rats were fed with standard diets supplemented with 0.5% (w/w) metformin as the positive blood sugar control (Metformin). The feeding procedures were maintained for 7 weeks.

### 2.4. Measurement of body weight and FBG level

The rats were weighed once per week. During the 7-week treatment, the FBG level was determined once a week using a glucose meter (Onetouch, LifeScan Inc.). The rats were fasted for 10 h before measurement, and blood was harvested using the tail-cutting method.

### 2.5. DNA isolation, library construction, and 16S rDNA amplicon sequencing

Bacterial DNA was extracted from the fresh intestinal content using a QIAamp PowerFecal DNA Kit (Qiagen), following the manufacturer's protocols. The quality and quantity of bacterial DNA were verified using a NanoDrop spectrophotometer and through agarose gel electrophoresis. Bacterial 16S rDNA amplicon (V3 and V4) regions were amplified with barcoded primers. The amplicons were purified using AMPure XP beads (Agencourt) and amplified for another round of PCR. The final amplicon was quantified using a Qubit dsDNA Assay Kit (Thermo Fisher), and equimolar concentrations of libraries were pooled and sequenced on an Illumina MiSeq platform at OE Biotech (Shanghai, China). The raw sequence reads were deposited in the NCBI Sequence Read Archive database (www.ncbi.nlm.nih.gov/sra/), with the accession number PRJNA926516.

### 2.6. Data processing and analysis

Paired-end reads were preprocessed using Trimmomatic software (version 0.35) to detect and filter ambiguous bases. The low-quality sequences with an average score below 20 were also cut off by sliding window trimming. The filtered reads were assembled using FLASH software (version 1.2.11) and denoised using QIIME software (version 1.8.0). The resulting clean reads were subjected to primer sequence removal and clustering to generate operational taxonomic units (OTUs) using Vsearch software (version 2.4.2) by a similarity cutoff of 97%. The representative read of each OTU was selected using the QIIME package. All representative reads were annotated and BLASTed against the Silva database (version 123) using the RDP classifier algorithm and a confidence threshold of 70%. The alpha diversities were measured using Chao1 richness, Simpson, and Shannon indexes. The OTU abundance table was used to analyze the beta diversity, including principal component analysis (PCA) and principal coordinate analysis (PCoA). The key communities of intestinal microbiota were determined using the linear discriminant analysis effect size (LEfSe) method. A *p*-value of < 0.05 and a linear discriminant analysis (LDA) score of ≥2.5 were considered statistically significant.

## 3. Results

### 3.1. An HSP-augmented diet lowers the FBG level of T2D model rats

An STZ injection was administered to induce T2D-like metabolic disease in SD rats. As expected, the STZ-injected rats exhibited glucose intolerance with an FBG level of >15 mmol/L 1 week after the first injection. These T2D model rats were grouped and subjected to different dietary processes (Figure 1C). Administration of the standard diet (Model) resulted in severe body weight loss and an extremely high FBG level (20 mmol/L) in T2D model rats, whereas this phenotypic change of T2D model rats could be effectively alleviated by dietary HSP augmentation (HSP 2.5 and HSP5.0). Especially, the FBG level lowered to below 10 mmol/L after 1 week of HSP-augmented feeding in both groups, albeit with a slight rebound at week 7. The analysis of variance (ANOVA) of the FBG observation after the first week indicated that HSP augmentation was superior to metformin administration (Supplementary Table 1, *P* = 0.0098), and Duncan's multiple range test showed that HSP at a concentration of 2.5% or 5.0% did not have a significant difference in controlling the FBG level of T2D model rats. In comparison with the Model group, the HSP 2.5 and HSP 5.0 groups, as well as the Metformin group, showed a slight increase in body weight. These results suggest that HSP augmentation potently alleviates high FBG levels in T2D model rats. The hypoglycemic effect of dietary HSP administration has also been reported in T2D model rats (Dong et al., 2020). Oral HSPs have been proven to regulate the gene expression involved in gluconeogenesis, lipid metabolism, and inflammation. We suspected the gut microbiota plays an essential role in bringing about these functions of HSP.

### 3.2. HSP augmentation induces a change in the gut bacterial composition in T2D model rats

**Figure 2:**
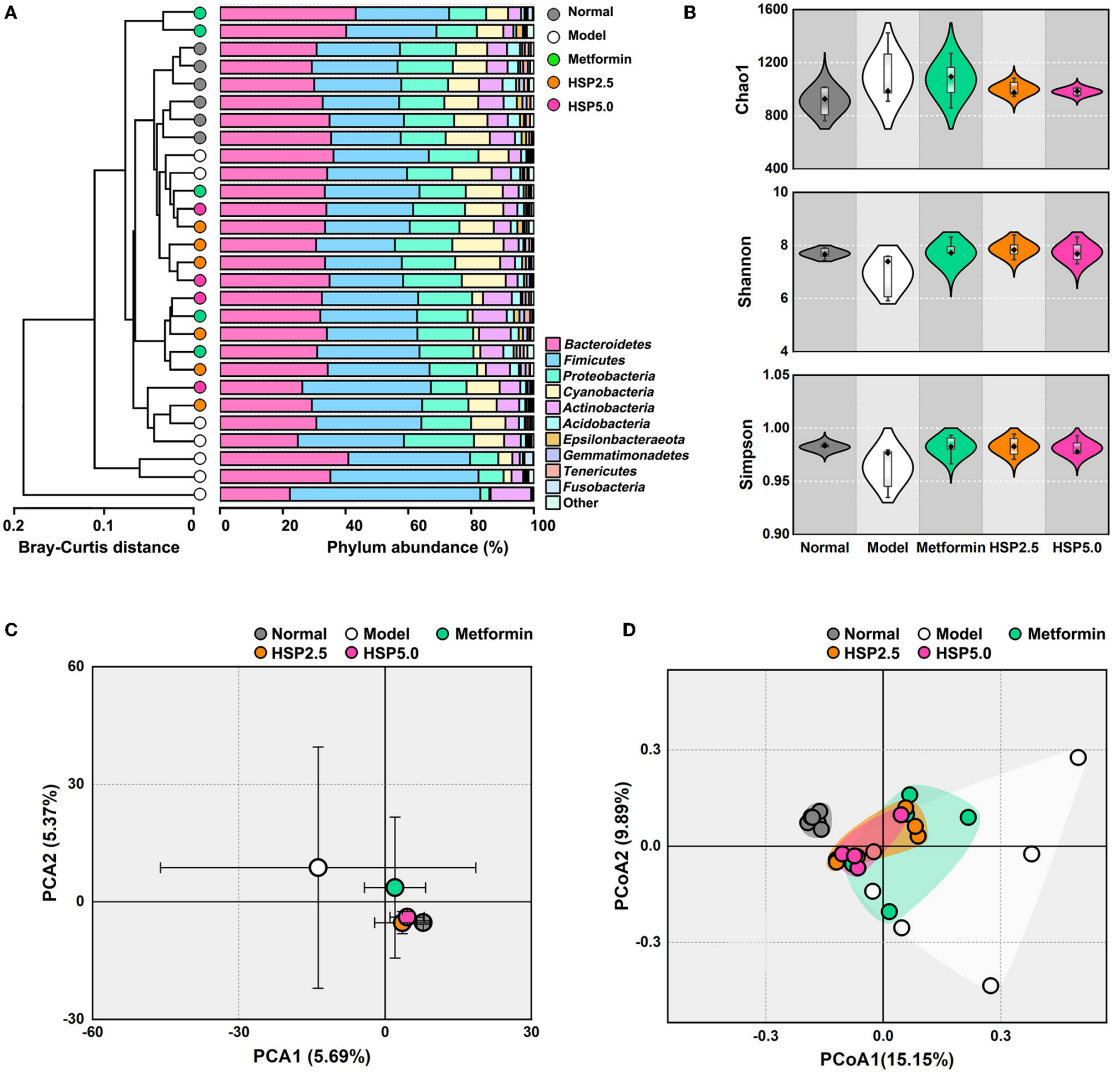
Analyses of alpha and beta diversities among groups. **(A)** Phylum structure of gut microbiota. The evolutionary tree was built with the Bray–Curtis distance using the UPGMA method. **(B)** Simpson, Shannon, and Chao indexes. **(C)** PCA. The error bars of each sample indicate the standard deviations at PC1 and PC2, respectively. **(D)** PCoA. The sample distributions are shadowed with different colors for each group.

To examine the effects of HSP augmentation on the intestinal microbiota of T2D model rats, the fecal samples were collected at the experimental endpoint (week 7) for sequencing of the 16S rRNA gene (Supplementary Table 2). As a result, an average of 76,762 valid reads (ranging from 70,331 to 81,207) was obtained for 28 samples. These quantified sequences were clustered into a total of 5,662 unique OTUs, and only 707 OTUs were shared in the five groups (Supplementary Figure 1, Supplementary Table 3), which suggested a difference in bacterial abundance among the groups. The composition overview at the phylum level showed that Bacteroidetes, Firmicutes, Proteobacteria, and Cyanobacteria are the major communities (>84%), and the Model group showed the highest profiling variation (Figure 2A), although the ANOSIM of Bray–Curtis distances did not show a significant difference (R = 0.083, *P* = 0.106) among the groups (Supplementary Figure 2). ANOVA (Supplementary Table 4) suggested a significant increase (*p* = 0.030) in the phylum Firmicutes in the Model group (38.5%) compared with the Normal group (25.3%) and groups administered other diets (28.8–30.6%), which contributed to the highest Firmicutes/Bacteroidetes (F/B) ratio in the Model group (1.29). These results suggest there were changes in gut microbiota in the T2D model rats. Thus, the alpha diversity metric was analyzed in all groups by measuring Chao1, Simpson, and Shannon indexes (Figure 2B). The results revealed that the bacterial species richness and diversity were significantly increased in the Model group compared with the Normal group. However, HSP administration either at 2.5 or 5% resulted in reductions in species richness and diversity of gut microbiota in T2D model rats, which was comparable to or better than the effects of metformin exposure. The PCA plot also suggested group differences between the Normal group, Model group, and HSP or metformin groups (Figure 2C). The Model group was clearly separated from the other groups, and the HSP or metformin dietary administration groups were close to the Normal group.

The overall microbial structure among the groups was further analyzed on the beta diversity metric using PCoA at the OTU level (Figure 2D), which suggested that HSP or metformin dietary administration can reshape the microbial structure of the Model group to a structure similar to that of the Normal group. A high microbiological variation (large standard deviation) was also observed among the T2D model rats. Nevertheless, these results demonstrate that HSP dietary administration contributes to a substantial shift in the gut microbiota of T2D model rats.

### 3.3. Identification of key taxa associated with an HSP-augmented diet in T2D model rats

**Figure 3:**
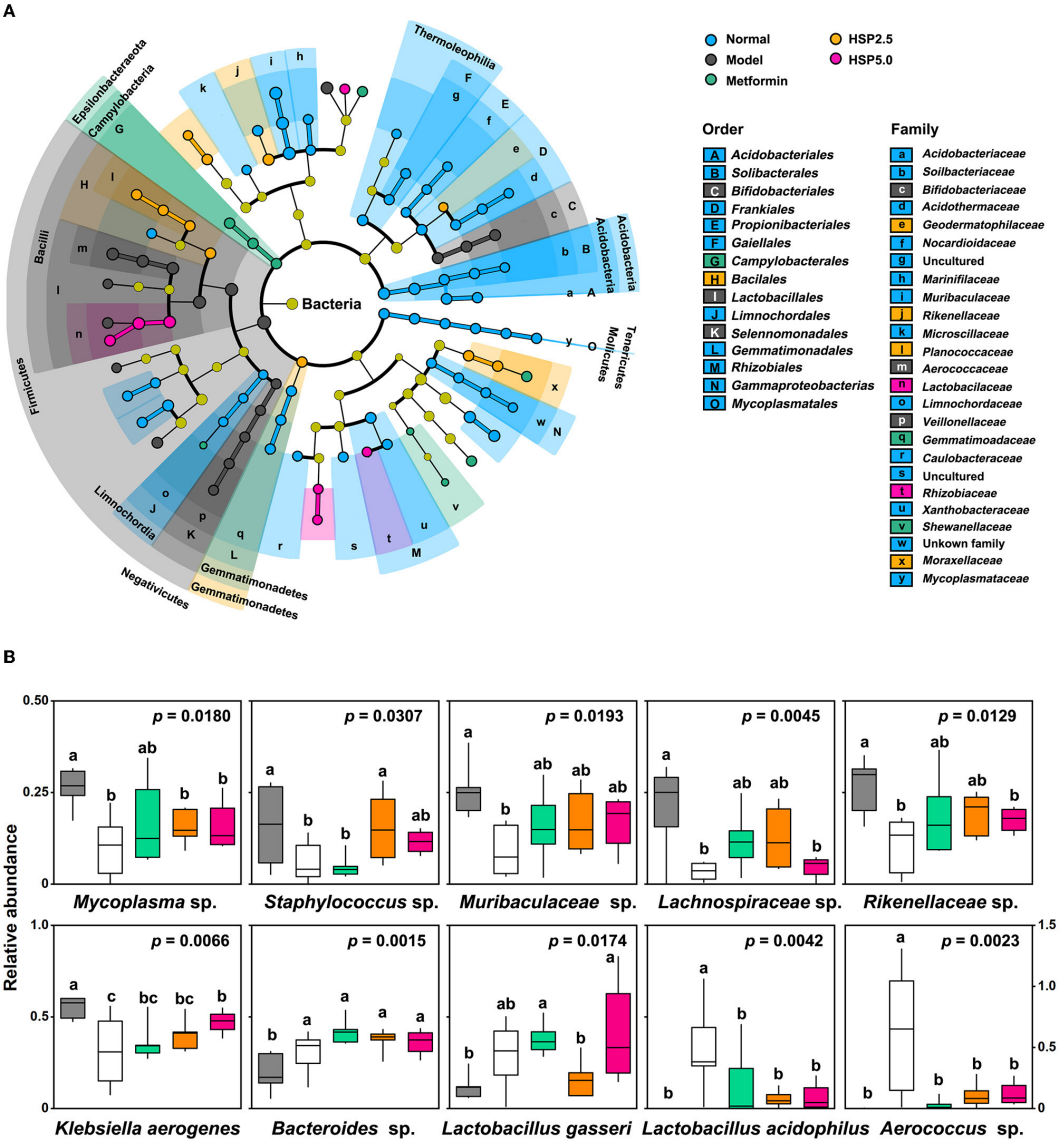
Taxa associated with an HSP-augmented diet. **(A)** LEfSe analysis. The analysis was performed using *P*=0.05 and a logarithmic LDA score of 3.0. **(B)** Relative abundances of the top 10 most significant OTUs. Relative abundances are presented as log_10_(100 × value + 1), and the *P*-values were adopted from ANOVA. Duncan's multiple range test is denoted by “a”, “b”, and ‘c' (*P* = 0.05).

Linear discriminant analysis (LDA) effect size (LEfSe) was employed to identify key bacterial taxa associated with an HSP-augmented diet using OTUs. The statistical differences in microbial communities were compared at different taxonomic levels, and taxa cladograms presented enrichment at a cutoff LDA score of >3 (Figure 3A, Supplementary Figure 3, Supplementary Table 5). Firmicutes was the dominant phylum in T2D model rats, which was consistent with the outcome of the highest F/B ratio in this group. The gut microbiota of the Normal group showed 15 predominant family cladograms out of 25 identified families; however, only the observed Limnochordaceae family belongs to the phylum Firmicutes. A similar observation was made at the genus level, in which three of 16 and five of six genera belong to the phylum Firmicutes for Normal and Model groups, respectively. Overall, these two groups showed a distinct distribution of enriched cladograms. Nevertheless, many enriched cladograms for the Normal group were also observed in the HSP 2.5, HSP 5.0, and Metformin groups (Figure 3A). This observation was consistent with the PCA analysis of microbial structure among the groups. It suggested that HSP dietary augmentation can change the composition of intestinal microbiota in T2D model rats as metformin performs.

**Figure 4:**
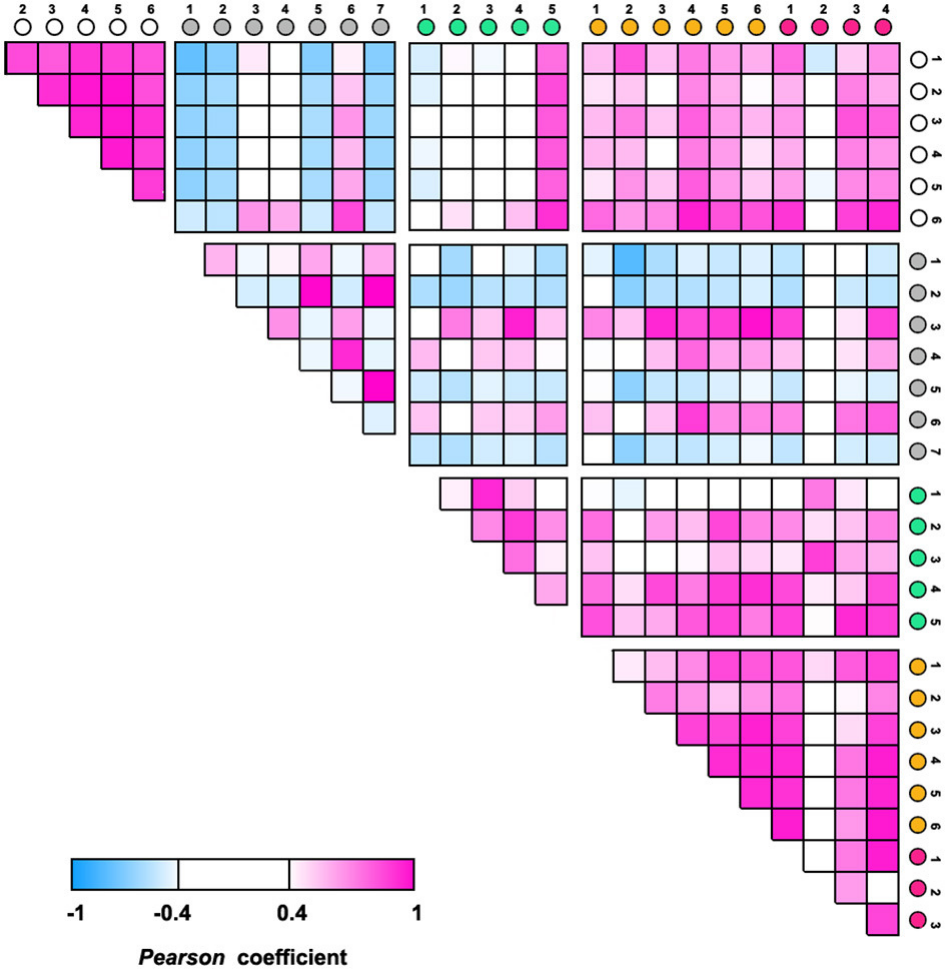
Pearson coefficient analysis among groups using the relative abundances of the top 10 significant OTUs.

ANOVA was performed to identify specific microbes associated with T2D disease and control using the relative abundance of OTUs. A total of 112 OTUs showed statistical differences (*p* < 0.05) among the five groups (Supplementary Table 6). They account for 35.1 ± 2.59% of the total abundance in the T2D Model group, 25.6 ± 7.01% in the Normal group, and 25.0–27.4% in the HSP- or Metformin groups. The top 10 most significantly changed species are presented in Figure 3B. *Mycoplasma* sp. (OTU160), *Staphylococcus* sp. (OTU170), *Muribaculaceae* sp. (OTU27), *Lachnospiraceae* sp. (OTU67), *Rikenellaceae* sp. (OTU84), and *Klebsiella aerogenes* (OTU22) had significantly higher abundances in normal rats than in the T2D model rats, whereas *Lactobacillus gasseri* (OTU), *Lactobacillus acidophilus* (OTU), and *Aerococcus* sp. (OTU) showed a reverse fashion between both groups. This distinct performance was elucidated through Pearson coefficient analysis (Figure 4), while the metformin and HSP administration could change the Model abundance toward those in the Normal group (Figures 3, 4). These abundance-increased OTUs in the Normal group are pathogens or opportunistic pathogens, which have often been reported to be increased in individuals with T2D (Redel et al., 2013; Zhang Z. et al., 2019; Deng et al., 2020). Such an aberrant appearance was most likely due to a huge increase in lactic acid bacteria (e.g., *L. gasseri* and *L. acidophilus*) and *Aerococcus* sp. Surprisingly, our results did not show the enrichment of some known SCFA-producing bacteria, such as *Clostridia*, in either the Metformin- or HSP-administered groups.

## 4. Discussion

Our study demonstrated the effect of lowering FBG levels through HSP administration, which even outperformed metformin (Figure 1C). Both HSP and metformin differentially affected the diversities and compositions of gut microbiota in T2D model rats. Diversity and richness were significantly increased in T2D model rats conpared with normal rats. Of interest, HSP administration reduced these alpha diversity indexes, which is often observed in drug administration in T2D animals (Lee et al., 2018; Yang et al., 2020). Additionally, HSP and metformin had differential effects on the composition of gut microbiota, according to beta diversity analysis (Figures 2C, D). HSP administration was more effective at restoring the diversity of normal microbiota than metformin in T2D model rats, whereas the dosage effect of HSP (either 2.5 or 5%) was not observed. Additionally, our results showed that HSP changes the F/B ratio in T2D model rats. It has been proposed that the F/B ratio is linked to T2D dysfunction and obesity, and SCFA metabolism is positively related to the F/B ratio (Fernandes et al., 2014; Wang and Jia, 2016). Our results contradicted the previous observation that individuals with T2D have a lower F/B ratio than individuals without diabetes (Demirci et al., 2020), which has remained controversial due to the experimental subjects (Schwiertz et al., 2010). As Firmicutes and Bacteroidetes were the most abundant phylogenetic categories, it was suspected that only a portion of bacterial species associated with SCFA metabolism contribute to T2D development. This change could not be directly assessed by the F/B ratio, while different F/B ratios could reflect the changes in gut microbiota. LEfSe analysis and ANOVA identified *L. gasseri* as an enriched species in T2D model rats (Figure 3). An increase in the abundance of *L. gasseri* has been reported in the T2D group of European women and correlates positively with fasting glucose and glycosylated hemoglobin HbA1c (Karlsson et al., 2013). *Lactobacilli*, including *L. gasseri*, are generally recognized as probiotics that can prevent obesity, improve glucose tolerance, and attenuate inflammation (Shirouchi et al., 2016; Niibo et al., 2019; Youn et al., 2021). Host species, enterotypes, different compounds, and interval of dietary administration probably caused the discrepancy among different cases. Nevertheless, *Lactobacilli* have been identified as a potential infectious factor in immunocompetent individuals (Pararajasingam and Uwagwu, 2017; Ramos-Coria et al., 2021). It would not be surprising to observe an increase in *L. gasseri* in T2D model rats with immune dysfunction. It should also noted that the 16S rDNA sequencing used in this study was limited to interpreting relevant function at a species level. The STZ induced diabetic model might be the same reason in human T2D. However, this study demonstrated that HSP administration was an effective way of controlling FBG levels in T2D model rats, and the beneficial effects were associated with changes in gut microbiota. Our results suggested a possible use of HSP dietary administration to build microbiota of T2D patients in clinical practice.

## Data availability statement

The original contributions presented in the study are included in the article/Supplementary material, further inquiries can be directed to the corresponding authors.

## Ethics statement

All animal experiments were conducted in accordance with the relevant regulations required by the International Animal Welfare Committee and the Animal Experiment Operation and Ethics Committee of Soochow University. The Institutional Review Board Statement and approval number for studies involving humans or animals. Approval Code: 201911A063, Approval Date: 5 November 2019.

## Author contributions

AY, XZ, CW, and Y-QZ designed the research and analyzed the data. WZ, YW, and QL conducted the research. WZ, XZ, and CW wrote the manuscript. AY, XZ, and WZ had primary responsibility for the final content. All authors read and approved the final manuscript.
