# Supplementary material for: Dietary administration with hydrolyzed silk sericin improves the intestinal health of diabetic rats
Source: Front Microbiol. 2023 Mar 7;14:1074892. doi: 10.3389/fmicb.2023.1074892 (PMC10027739; doi:10.3389/fmicb.2023.1074892)
Supplement: Supplementary file 1 [file Data_Sheet_1.docx]

Supplementary Material

# Supplementary Figures and Tables

**Table S1.** ANOVA analysis of FGB levels among Metformin, HSP2.5 and HSP5.0 groups.

**Table S2.** The absolutely abundance table of OTUs by 16S rRNA sequencing.

**Table S3.** Statistic description of 16S RNA sequencing for samples

**Table S4.** ANOVA analysis of relative abundance of Firmicutes and Bacteroidetes.

**Table S5.** The core taxa determined by linear discriminant analysis (LDA) effect size (LEfSe).

**Table S6.** ANOVA analysis of relative abundance of OTUs.

**Figure S1.** Venn diagram of the identified OTUs among groups.

**Figure S2.** Anosim analysis of Bray-Curtis distances among groups.

**Figure S3.** The taxa changes associated with HSP dietary administration determined by linear discriminant analysis (LDA) effect size (LEfSe)

**Table S1. ANOVA analysis of FGB levels^a^ among Metformin, HSP2.5 and HSP 5.0 groups.**

| Sources | SS | df | MS | F | P-value |
| --- | --- | --- | --- | --- | --- |
| Between | 87.23 | 2 | 43.61 | 6.04 | 0.0098 |
| Group | 129.98 | 18 | 7.22 |  |  |
| Total | 217.21 | 20 |  |  |  |

^a^ FGB levels were adopted measurements from the first week, and the means of FGB levels at each week was used for ANOVA analysis.

**Table S3. Statistic description of 16S RNA sequencing for samples.**

| **Samples** | **Normal** | **Model** | **Metformin** | **HSP2.5** | **HSP5.0** |
| --- | --- | --- | --- | --- | --- |
| Sample counts | 6 | 7 | 5 | 6 | 4 |
| Valid tags | 6,566-81,207 | 71,293-79,549 | 70,331-77,468 | 70,594-78,352 | 73,822-79,711 |
| Valid percentage (%) | 87.8-91.59 | 84.2-92.0 | 85.1-92.3 | 85.9-93.0 | 86.6-91.0 |
| Valid mean length (bp) | 411.6 | 413.5 | 411.1 | 411.5 | 412.8 |
| OUT counts | 609-812 | 823-1323 | 727-1149 | 776-934 | 821-867 |
| Total unique OTUs | 2131 | 3192 | 2532 | 2527 | 1998 |

**Table S4. ANOVA analysis of relative abundance^a^ of *Firmicutes* and *Bacteroidetes*.**

| **Sources** | ***SS*** | ***df*** | ***MS*** | ***F*** | ***p*-value** |
| --- | --- | --- | --- | --- | --- |
| **Firmicutes** |  |  |  |  |  |
| Between | 228.80 | 4 | 57.20067 | 3.24 | 0.030 |
| groups | 406.66 | 23 | 17.68081 |  |  |
| Total | 635.46 | 27 |  |  |  |
| **Bacteroidetes** |  |  |  |  |  |
| Between | 22.26 | 4 | 5.57 | 0.71 | 0.595 |
| groups | 180.88 | 23 | 7.86 |  |  |
| Total | 203.14 | 27 |  |  |  |

^a^ Relative abundance was conducted with arcsine transformation before ANOVA analysis.
