# Supplementary figures and images for: Dietary administration with hydrolyzed silk sericin improves the intestinal health of diabetic rats
Source: Front Microbiol. 2023 Mar 7;14:1074892. doi: 10.3389/fmicb.2023.1074892 (PMC10027739; doi:10.3389/fmicb.2023.1074892)

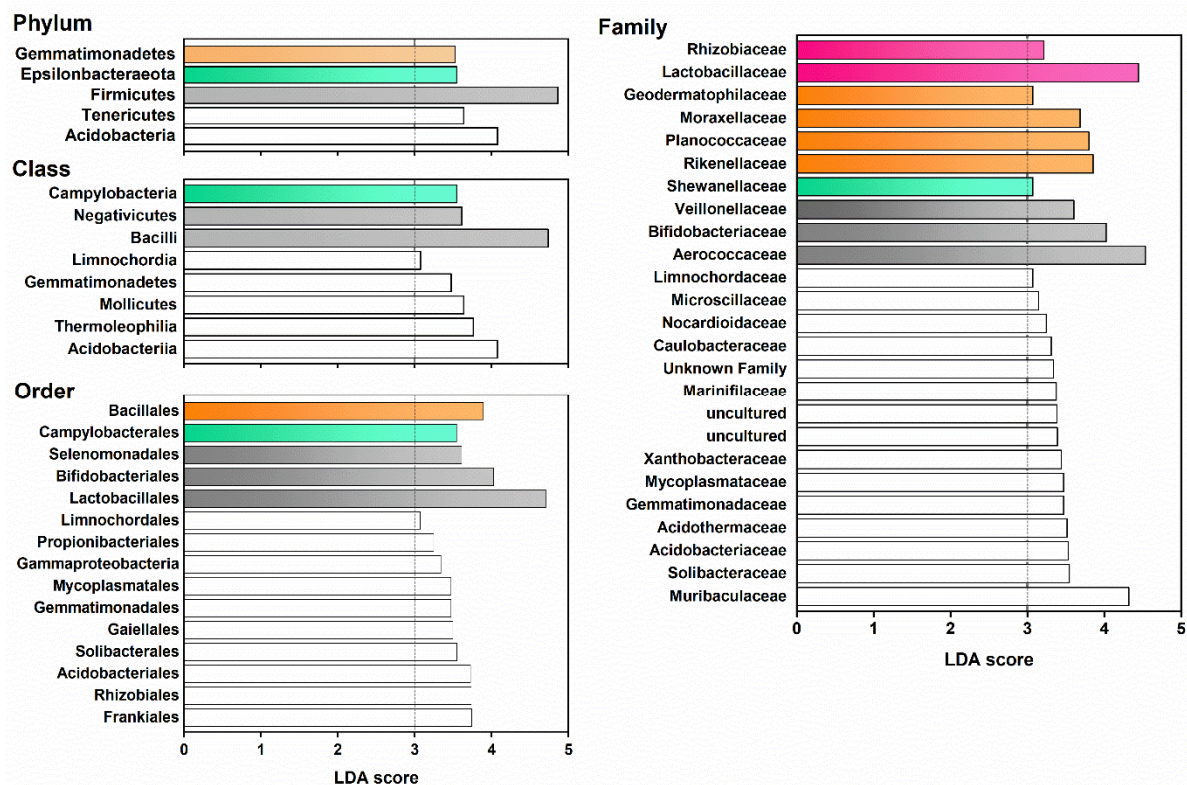

Supplement: Supplementary file 4 [file Image_2.PDF]

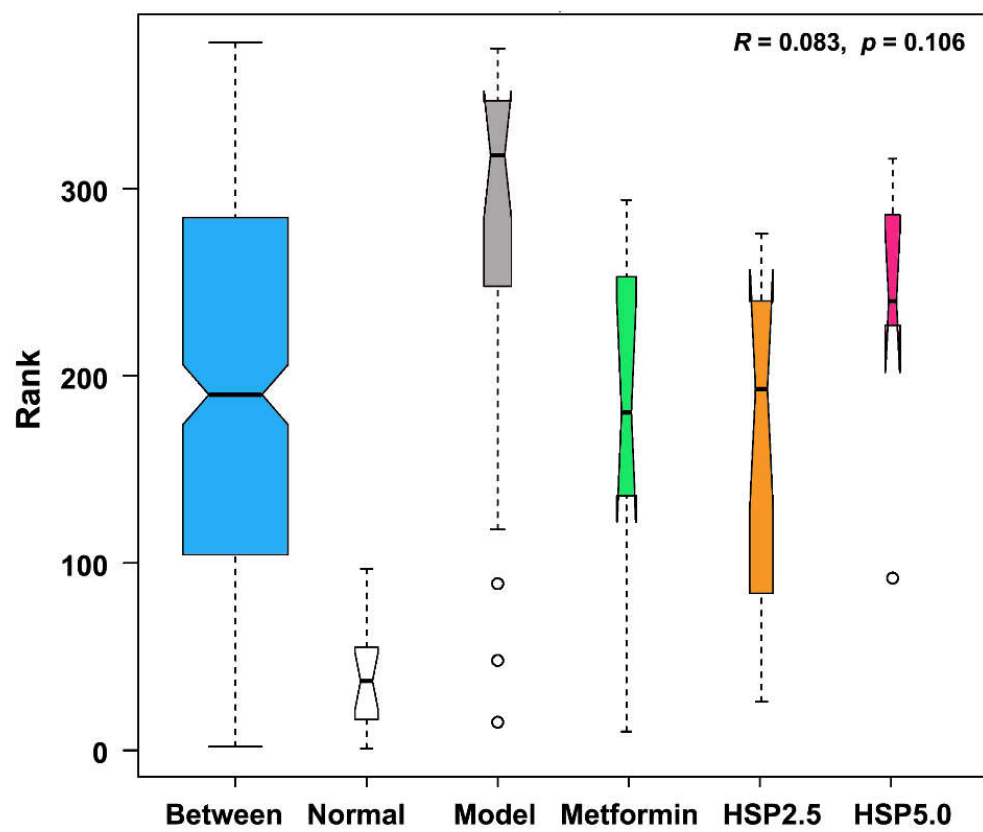

Supplement: Supplementary file 5 [file Image_3.PDF]
