# Supplementary material for: Dietary administration with hydrolyzed silk sericin improves the intestinal health of diabetic rats
Source: Front Microbiol. 2023 Mar 7;14:1074892. doi: 10.3389/fmicb.2023.1074892 (PMC10027739; doi:10.3389/fmicb.2023.1074892)
Supplement: Supplementary file 3 [file Image_1.PDF]

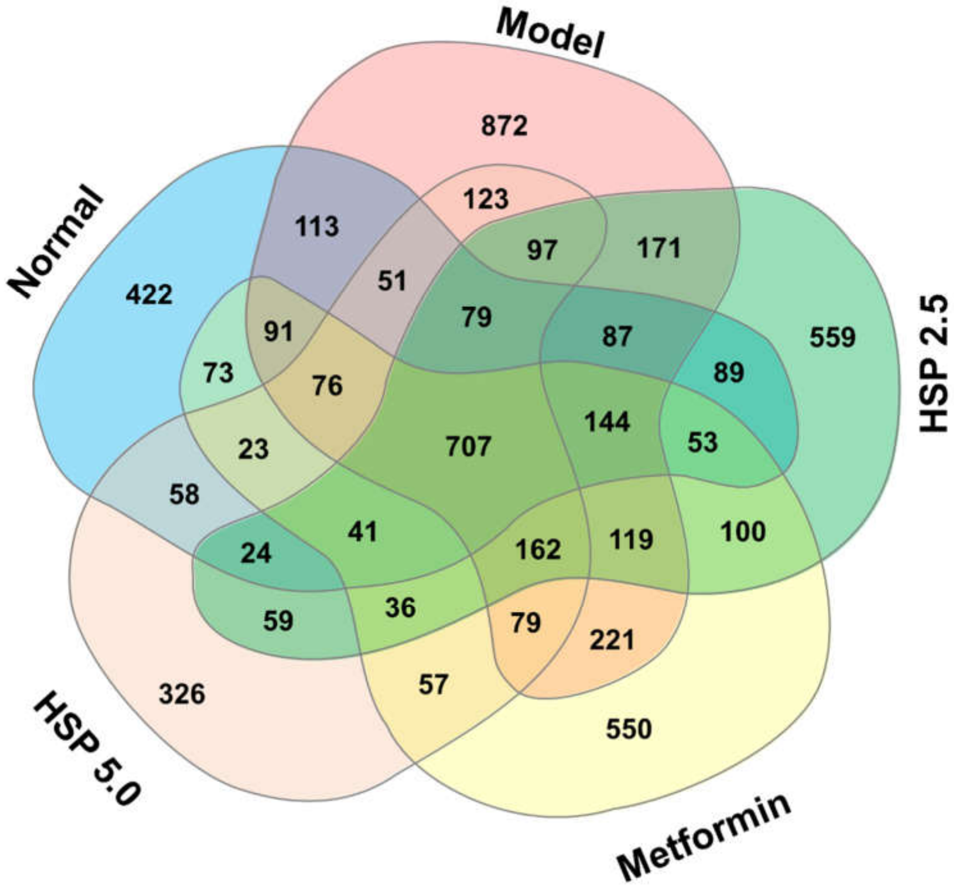

Model
Normal
HSP 2.5
HSP 5.0
Metformin
872
123
113
97
171
51
422
79
87
559
91
73
76
89
707
144
23
53
58
41
100
24
162
119
59
36
79
221
326
57
550
